# SiRNA Inhibits Replication of Langat Virus, a Member of the Tick-Borne Encephalitis Virus Complex in Organotypic Rat Brain Slices

**DOI:** 10.1371/journal.pone.0044703

**Published:** 2012-09-12

**Authors:** Carola Maffioli, Denis Grandgirard, Stephen L. Leib, Olivier Engler

**Affiliations:** 1 Neuroinfection Laboratory, Institute for Infectious Diseases, University of Bern, Bern, Switzerland; 2 Biology Division, Spiez Laboratory, Federal Office for Civil Protection, Spiez, Switzerland; Universidade Federal do Rio de Janeiro, Brazil

## Abstract

Tick-borne encephalitis virus is the causative agent of tick-borne encephalitis, a potentially fatal neurological infection. Tick-borne encephalitis virus belongs to the family of flaviviruses and is transmitted by infected ticks. Despite the availability of vaccines, approximately 2000–3000 cases of tick-borne encephalitis occur annually in Europe for which no curative therapy is available. The antiviral effects of RNA mediated interference by small interfering RNA (siRNA) was evaluated in cell culture and organotypic hippocampal cultures. Langat virus, a flavivirus highly related to Tick-borne encephalitis virus exhibits low pathogenicity for humans but retains neurovirulence for rodents. Langat virus was used for the establishment of an *in vitro* model of tick-borne encephalitis. We analyzed the efficacy of 19 siRNA sequences targeting different regions of the Langat genome to inhibit virus replication in the two *in vitro* systems. The most efficient suppression of virus replication was achieved by siRNA sequences targeting structural genes and the 3′ untranslated region. When siRNA was administered to HeLa cells before the infection with Langat virus, a 96.5% reduction of viral RNA and more than 98% reduction of infectious virus particles was observed on day 6 post infection, while treatment after infection decreased the viral replication by more than 98%. In organotypic hippocampal cultures the replication of Langat virus was reduced by 99.7% by siRNA sequence D3. Organotypic hippocampal cultures represent a suitable *in vitro* model to investigate neuronal infection mechanisms and treatment strategies in a preserved three-dimensional tissue architecture. Our results demonstrate that siRNA is an efficient approach to limit Langat virus replication *in vitro*.

## Introduction

Tick-borne encephalitis virus complex represents a group of closely related viruses endemic in Europe and Asia causing serious neuroinfections and hemorrhagic fevers [1]. Tick-borne encephalitis virus (TBEV) belong to the *Flaiviridae* family and can be divided in three main subtypes, the European, the Siberian and Far Eastern subtypes [2]. TBEV are transmitted to humans by the bite of infected ticks and, in rare cases, through the consumption of infected unpasteurized milk [3], [4]. Following an incubation period of 3–8 days after a tick bite, the virus replicates locally in epidermal dendritic cells and spreads via lymph vessels to the blood stream where a short but significant viremia occurs and extraneural tissues are infected. It is during this viremic phase that the virus crosses the blood-brain barrier and invades the central nervous system (CNS) by a still unknown mechanism. Once the virus has reached the CNS, active infection causes inflammation, lysis of cells and cellular dysfunction [5], [6]. Clinical manifestations of TBEV infections typically follow a biphasic course. The first viremic phase is characterized by flu-like symptoms such as fever, headache and muscle pain. After 3–7 days of an asymptomatic phase, 20–30% of the patients develop the second meningoencephalitic phase with neurological disorders of varying severity [5], [7], [8], [9]. The most common clinical feature of TBE patients is ataxia followed by paresis or paralysis of one or more extremities [10].

Two efficient formalin-inactivated whole virus vaccines are available in Europe and provide a high degree of protection against the disease. Nevertheless more than 2500 TBE cases have been registered yearly in Europe in the past ten years and a continuous increase in TBE morbidity was observed in the last years [11], [12]. Up to today no specific therapeutic options are available for flaviviral infections and an effective therapy for TBEV infection would be highly desirable [13]. Results from *in vitro* and *in vivo* studies indicate that therapeutics based on RNA interference (RNAi) could be effective against viral infections, and small interfering RNA (siRNA) molecules are promising candidates for future clinical applications [14], [15], [16], [17]. Indeed, several RNAi-based antiviral drugs are currently being tested in clinical trials [18], [19], [20], [21].

RNA interference is a conserved post-transcriptional gene silencing process which leads to the specific degradation of RNA within the cytoplasm of eukaryotic cells [22], [23], [24]. Long double-stranded RNA molecules are initially processed by the enzyme Dicer into 21–25 nucleotides long small interfering RNA. In the cytoplasm siRNAs are incorporated into the multiprotein RNA-induced silencing complex (RISC) which results in sequence specific association and degradation of the complementary mRNA [24], [25]. SiRNA mediated intervention strategies take advantage of this conserved mechanism by artificially introducing siRNA molecules into the cytoplasm which ultimately leads to the degradation of target RNA molecules. It was shown that the application of siRNA molecules with sequences complementary to viral genomic RNA or RNA replication intermediates allows to significantly reduce the number of virus progeny in infected cells. Using this strategy the replication of human pathogenic flaviviruses such as West Nile virus (WNV), Dengue virus (DENV) and Japanese encephalitis virus (JEV) was successfully inhibited both *in vitro* and *in vivo* [14], [26], [27], [28], [29], [30] and recently inhibition of TBEV by RNAi was demonstrated in human embryonic kidney cells (HEK293T) [31]. Sensitive target sequences were identified within the structural (Capsid (C), membrane protein (M) and envelope (E)), non-structural (mostly NS5 and NS3) and the 3′ untranslated region of the flavivirus 11 kb open reading frame (ORF).

We used organotypic hippocampal brain slice cultures (OHCs) infected with Langat virus (LGTV) as a model to analyze the effectiveness of RNAi in the inhibition of the virus replication. OHCs is a well established model based on *in vitro* cultured brain slices which was adapted for this virus encephalitic model from previous work [32]. Organotypic cultures offer unique advantages over other *in vitro* models, in that they reproduce important aspects of the *in vivo* situation. Organotypic cultures retain the three-dimensional tissue architecture of the brain with a preserved cellular composition including neurons, microglial cells and astrocytes [32], [33]. The use of OHCs allowed the culture of Langat virus in an *in vitro* system reproducing some aspects of TBEV infection of the brain. LGTV is a member of the tick-borne encephalitis virus complex sharing high nucleotide homology with TBEV. It has an attenuated virulence for the human host and was used as a vaccine candidate against TBE for several years, but was abandoned due to the sporadic occurrence of encephalitis cases (1∶18000) [34], [35]. Because of its relative avirulence for humans it can be handled under BSL-2 conditions. LGTV retains neurovirulence for rodents and is therefore considered a suitable virus-model for the pathogenic TBEV in mice and rats [36], [37], [38], [39]. Using Langat virus in the organotypic brain culture model we could demonstrate the effectiveness of siRNA in inhibiting TBE virus replication in a three-dimensional neuronal tissue architecture.

## Results

### Evaluation of siRNA Sequences Targeting Langat Virus Genome

**Figure 1:**
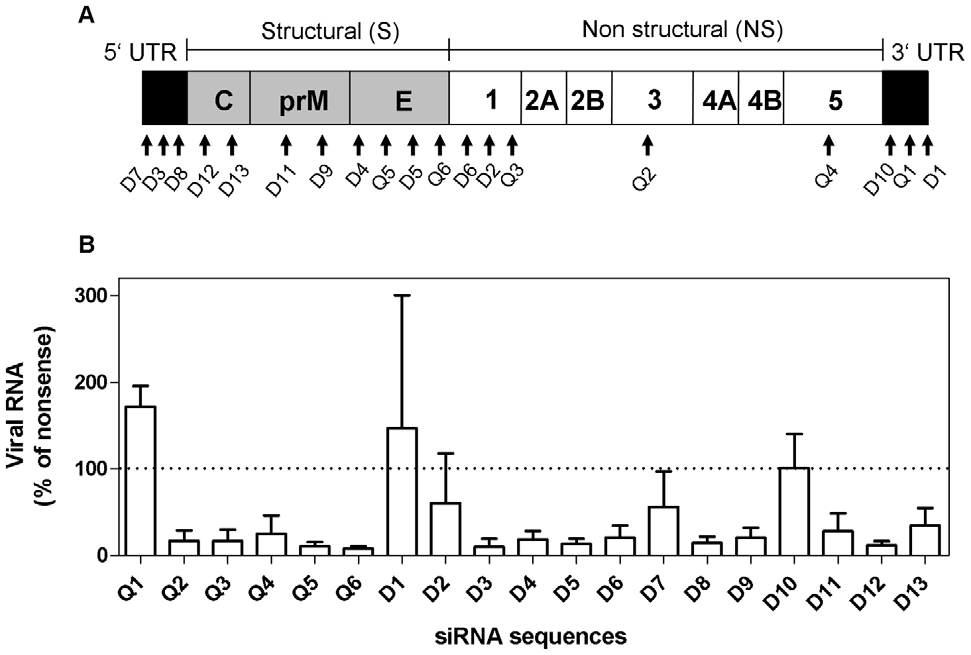
LGTV-specific siRNA sequences inhibit production of viral RNA in HeLa cells. Nineteen siRNA sequences (Q1–Q6 and D1–D13) targeting genes within the whole open reading frame of LGTV genome were analyzed for their antiviral potential on HeLa cells (A). After transfection with siRNA, cells were infected with Langat virus (MOI = 10) and six days later virus replication was assessed by quantitative real-time RT-PCR (B). Results are shown as a percentage of virus inhibition compared to the control cells transfected with the non-coding siRNA. Data are presented as mean ± SD of three independent experiments. Fourteen (Q2, Q3, Q4, Q5, Q6, D3, D4, D5, D6, D8, D9 D11 D12 and D13) out of 19 were significantly reduced (p<0.05) compared to a theoretical mean of 100% expressed by cells treated with nonsense siRNA; measured by One sample t test.

A total of 19 siRNA sequences (Q1–Q6 and D1–D13) targeting genes within the structural (S), the non-structural (NS) and the 3′ and 5′ untranslated regions (UTRs) of Langat genome (Table 1 and Figure 1 A) were tested. All siRNA sequences were evaluated for their capacity to inhibit replication of Langat virus in HeLa cells when transfected 4 h before the infection occurred. Fourteen out of the 19 specific siRNA sequences were able to induce a significant reduction of viral genome copy numbers in comparison to the nonsense siRNA used as a negative control (Figure 1B).

**Table 1:** Antisense sequences and positions of siRNAs used for inhibition.

Name	Sequence	Nt Position (5′ –3′)	Genome region
Q1	5′ – UAUAACGCCCAGUUCGGCCUU –3′	10730	3′UTR
Q2	5′ – UUGACGGAACGAACCAGGCUG –3′	5688	NS3
Q3	5′ –UUGAGUUCACUACUCCGGCAU–3′	3159	NS1
Q4	5′ – UAGUAUGACCAGCCGCCUCUG –3′	7912	NS5
Q5	5′ – UUAGAUGAUACUUAGUUCCCT –3′	1800	Env
Q6	5′ – UCUGAUGACACUGUGAACGAG –3′	1459	Env
D1	5′ – UUUCUCUCUUCCCUCCUCCUU –3′	10777	3′UTR
D2	5′ – UCAUCCACAGACUUUGAUCUU –3′	3047	NS1
D3	5′ – UUUCUCAACACGUUCACGAUU –3′	83	5′UTR
D4	5′ – AUGCUCAUGUGUCUUGUCCUU –3′	1576	Env
D5	5′ – UUCUGCAAGGCCUAGUUCCUU –3′	1970	Env
D6	5′ – UUCCACUCCAAUCAUGAACUU –3′	2857	NS1
D7	5′ – AUAUCCACAAUCACAGUCGUU –3′	47	5′UTR
D8	5′ – AUUCCCAGCUCUUGUUCUCUU –3′	114	5′UTR
D9	5′ – AAUCCAUCCACCAUCAACCUU –3′	897	pre M
D10	5′ – UUUCCCGUCACCACUCUCAUU –3′	10651	3′UTR
D11	5′ – UCUCCCUCGAGCCAUUGGUUU –3′	795	pre M
D12	5′ – UUUAGAACGGCCUUCCCGGUU –3′	135	Core
D13	5′ – AAGUCCAUUUGGCAUUUGGUU –3′	223	Core

Inhibition of virus replication was most efficient with siRNA molecules targeting sequences located within the structural region (Q5, Q6, D5, D12) and the 5′ UTR (D3 and D8), reducing viral genome copy numbers by up to 85%. Only Q1, D1 and D10, all targeting the 3′ UTR, had no effect on virus replication. A comparative analysis of all siRNA target sequences with the genomes of five members of the Tick-borne encephalitis virus complex revealed that siRNA sequences D1, D3, D8, D10 and D13 located within the 3′UTR, 5′UTR and the core region were strongly conserved with 1 to 2 nucleotide divergence between different viral subtypes. SiRNA sequence D3 was the only one with 100% sequence identity in all three TBEV subtypes and in the Omsk hemorrhagic fever virus (Table S1). SiRNA sequence D3 targeting 5′UTR was selected for further experiments on HeLa cells and organotypic cultures.

### Inhibition of Virus Replication by siRNA D3 in HeLa Cells

**Figure 2:**
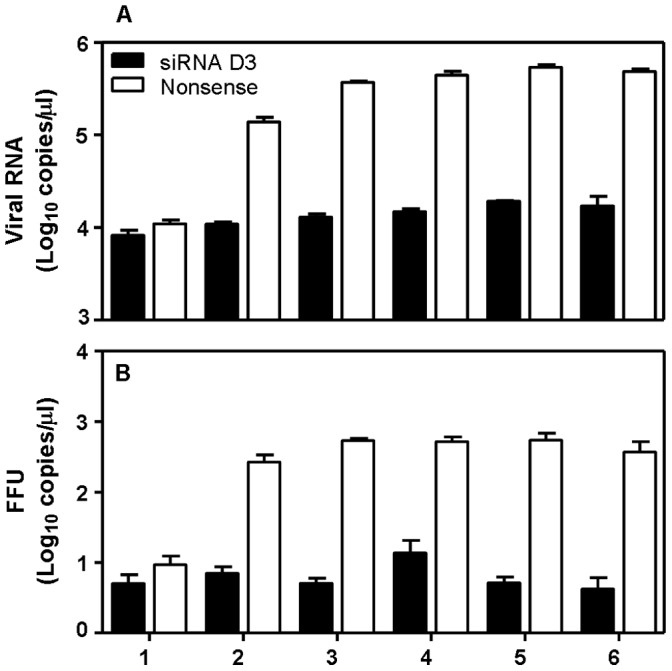
Inhibitory effects of siRNA D3 on viral replication on HeLa cells over time. The replication profile for Langat virus was determined for the time period of 6 days. Indicated are the numbers of RNA copies (A) and the number of infectious particles (B) for cell cultures treated with nonsense siRNA or specific siRNA D3.

To determine the inhibitory effect of siRNA sequence D3 over time the replication profile of Langat virus was assessed daily during the 6 days incubation period in cells treated with specific siRNA D3 and in cells treated with nonsense siRNA as controls. While virus replication was continuous in HeLa cells treated with nonsense siRNA, the number of viral genome copies was reduced by 96.5% (1.4 logs) (Figure 2A) and the number of infectious particles by 98.8% (2 logs) (Figure 2B), from day 2 to day 6 post infection in cells transfected with siRNA D3. To exclude unspecific inhibition of virus replication trough an effect of the transfection reagent on cell viability we performed a LDH-based cytotoxicity assay on cells under the different treatment regimes. No significant difference in cytotoxicity was observed between transfected and infected cells and untransfected and uninfected cells demonstrating that cell viability was comparable between the four groups (Figure S1).

**Figure 3:**
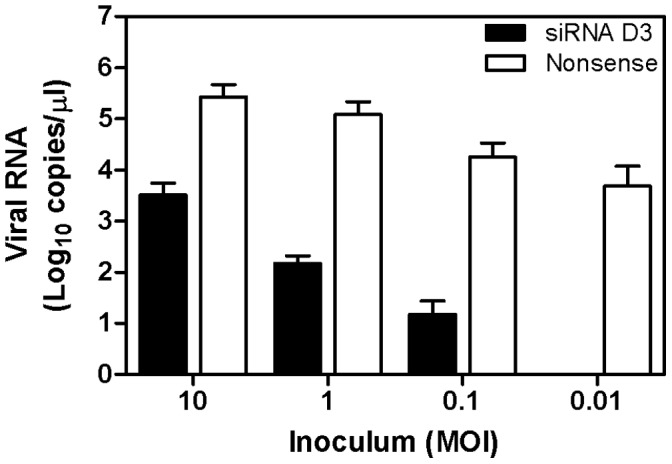
Antiviral effect of siRNA D3 in relation to the infectious dose of Langat. HeLa cells were infected with Langat virus at different infectious dose (MOI of 10, 1, 0.1 or 0.01) and one hour after infection, cells were treated with 200 nM siRNA sequence D3 or with nonsense siRNA. Virus titer was assessed by quantitative real-time RT-PCR 6 days after infection in the cell culture supernatant. The reduction of viral RNA in siRNA D3 treated cells is statistically significant compared to nonsense siRNA treated cells for all four MOI (p<0.05). The data are presented as mean ± SD of three independent experiments and significance calculated using an unpaired T-test.

We analyzed the effectiveness of siRNA on viral replication when administered after the initiation of the infection. To this end HeLa cells were first infected with Langat virus at MOI of 10, 1, 0.1 or 0.01 and 1 h later transfected with siRNA sequence D3. Compared to cells transfected with the nonsense siRNA sequence, treatment of infected HeLa cells with siRNA sequence D3 resulted in a reduction of viral genome copies by 1.6 log (MOI of 10), 2.4 log (MOI of 1), 2.4 log (MOI = 0.1) and 5 log (MOI = 0.01), respectively which corresponds to an inhibition of more than 98% for all the infectious doses. At the lowest infectious dose of 0.01 MOI the virus titer was reduced below the detection limit (Figure 3).

### Langat Virus Replication in the Organotypic Hippocampal Cell Culture Model

**Figure 4:**
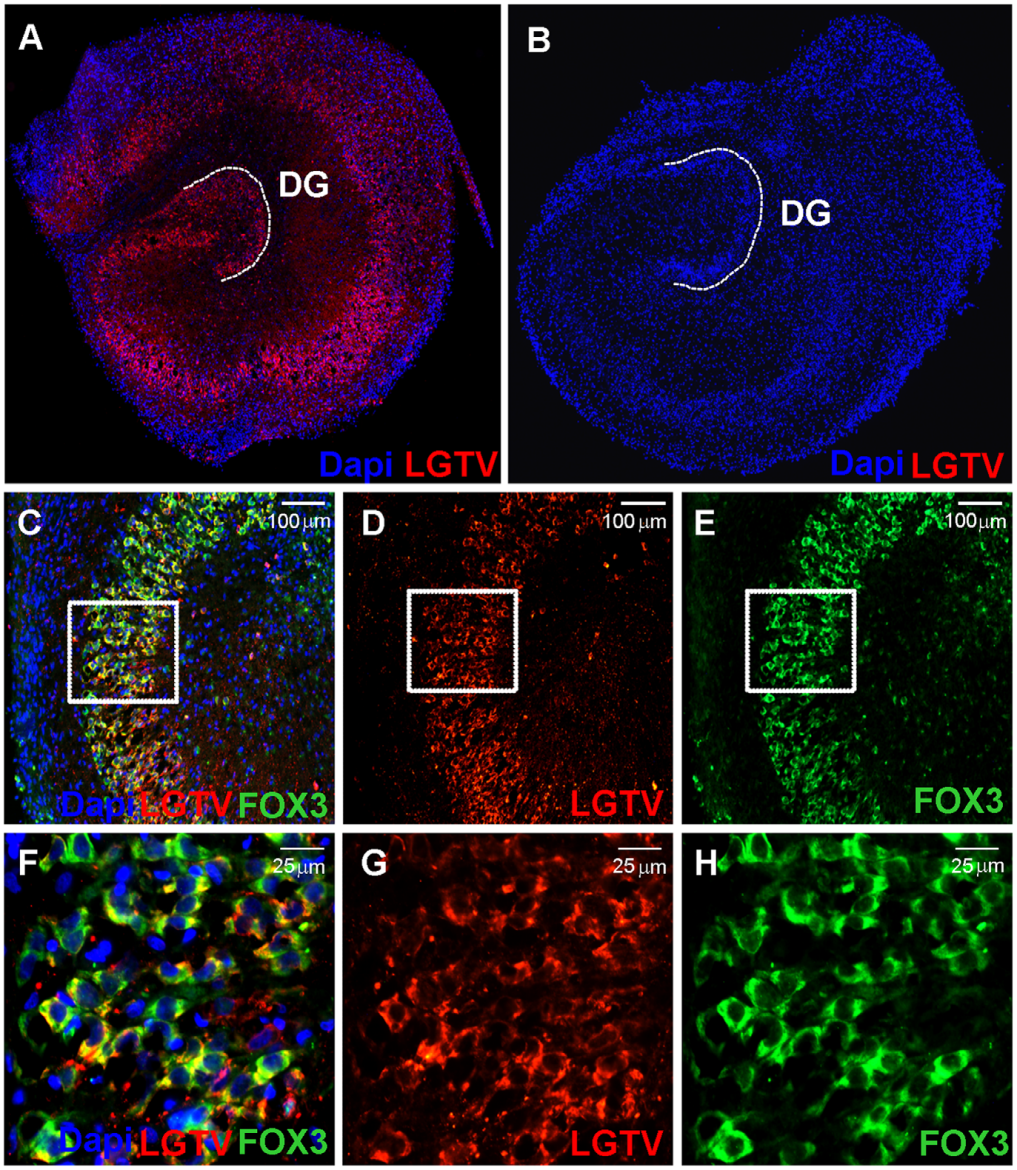
Expression of Langat virus proteins in infected organotypic hippocampal cultures. OHCs were infected with 2×10E6 FFU Langat virus for 7 days and immunostained with an anti-Langat virus antibody (LGTV; red) (A). Uninfected slices are shown as a control (B). Double staining of viral proteins (LGTV; red) (D 10×; G 40×) and neurons (FOX3; green) (E 10×; H 40×) on infected OHCs showed colocalisation (C 10×; F 40×). Cell nuclei were counterstained with Dapi (blue).

OHCs are an excellent model for the investigation of neuronal infection mechanisms and new treatment strategies. The ability of Langat virus to infect and replicate in OHCs was demonstrated by real-time RT-PCR and immunofluoresence (Figure 4). Using a red fluorescent antibody, cells expressing Langat virus proteins were specifically stained. Figure 4 A shows a strong expression pattern of viral proteins in OHC infected with Langat virus in contrast to uninfected OHC depicted in Figure 4 B. Co-staining of Langat virus proteins (red) and Fox 3 (green), a marker for neuronal cells, shows strong co-localisation, especially in the cornu ammonis (CA) and dentate gyrus (DG) (Figure 4 C, D, E, F, G, H).

### Antiviral Activity of siRNA in Infected Organotypic Hippocampal Cell Cultures

**Figure 5:**
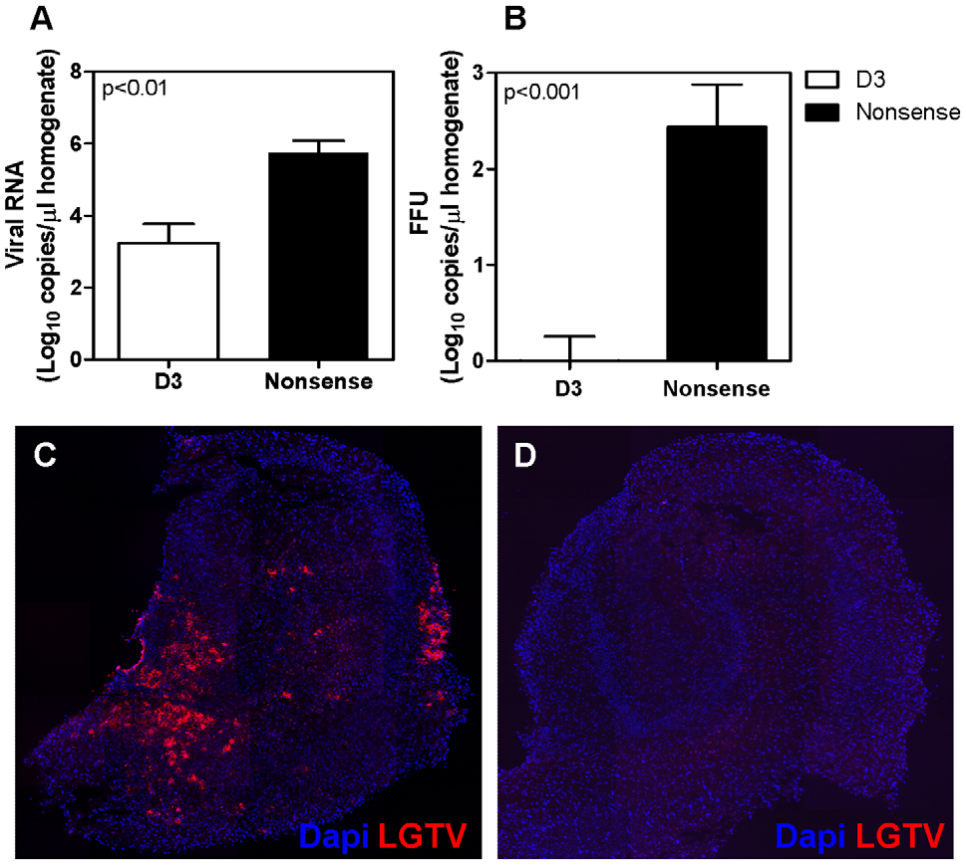
Antiviral effect of RNAi on rat organotypic hippocampal cultures. OHC were transfected with 800 nM specific siRNA D3 or with nonsense siRNA 24 h before and 1 h after the infection with LGTV. OHC were incubated for 6 days and virus titer assessed. The number of genome copies (A) and the number of infectious particles (B) measured in OHCs pre- and posttreated with siRNA D3 or nonsense siRNA are indicated. Expression of viral proteins was analyzed by immune staining of Langat virus proteins (red) in microtome slices of OHCs treated with specific siRNA D3 (D) or with nonsense siRNA (C). Cell nuclei were stained with Dapi (blue). Figures C and D are representative images of six independent experiments.

To assess the capacity of siRNA to inhibit virus replication in a complex three-dimensional neuronal network, inhibition experiments were performed on OHCs. Transfection of OHCs with the specific siRNA sequence D3 before and after the infection led to an efficient inhibition of virus replication reducing the number of viral genome copies in OHC homogenates from 5.4×10E5 to 1.7×10E3 copies/µl (2.7 logs) and the number of infectious particles from 2.7×10E2 to an average of 0.5 ffu/µl (4.3 logs) (Figure 5 A and B) corresponding to a reduction of more than 99.6% as compared to nonsense siRNA treated OHCs. No difference in cytotoxicity was observed by assessing LDH release between transfected OHCs and controls (Figure S2). The marked reduction of viral progeny in organotypic cultures treated with siRNA was confirmed by immunostaining methods. While in OHCs transfected with the non-targeting siRNA the expression of viral proteins led to strong red fluorescent signal in a high percentage of the cells, in OHCs transfected with the specific siRNA sequence D3 no red fluorescence was detectable, confirming an efficient reduction of viral protein levels in all cells (Fig. 5 C and D).

### Off-target Effects

Several studies suggest that transfection of cells with siRNAs may result in the activation of the interferon pathway and affect virus replication via interferon mediated antiviral activity [40], [41], [42]. To investigate whether the treatment of OHCs with siRNA leads to the activation of the IFN system, expression levels of IFN-β mRNA were quantified by real time RT-PCR in non-infected OHCs treated with different siRNA sequences and compared to untreated or poly I:C treated cultures. While treatment of HeLa cells with poly I:C, used as a positive control, leds to a strong upregulation of IFN-β mRNA, none of the siRNA sequences lead to an increase in the IFN-β mRNA expression 24 h after the second transfection (Figure S3).

## Discussion

Therapeutic options for the treatment of TBE are lacking and specific therapies are urgently needed since the incidence of TBE is rising. Several studies have shown that siRNA molecules have the potential to be used as a specific therapeutic strategy against viral infections. Due to difficulties in delivery, toxicity and the stimulation of unspecific immune response few approaches were continued *in vivo*. For TBE virus infections detailed investigations determining optimal target sequence and inhibitory capacity for synthetic siRNA are lacking. The majority (11/19) of the 19 siRNA sequences screened in this study were capable of reducing Langat virus titer by more than 80% in comparison to nonsense siRNA when transfected into the cells before the infection (Figure 1 B). The most effective reduction in viral replication was achieved with siRNA sequences targeting the 3′ UTR and the structural genes. SiRNA sequence D3 (5′UTR) and Q6 (Envelope) showed the highest capacity to inhibit viral replication, reducing the number of viral RNA genome copies from 1×10E6/µl (nonsense siRNA) to 5.5×10E4/µl (siRNA D3) and 6.6×10E4/µl (siRNA Q6) respectively, corresponding to a reduction of more than 93% (Figure 1 B). In addition to its strong antiviral activity sequence D3 is highly conserved between different members of the tick-borne encephalitis virus complex and was therefore selected for further experiments on HeLa cells and organotypic brain cultures. Using the siRNA sequence D3 we could demonstrate that the application of siRNA after the infection was still effective. In fact the inhibitory effect was comparable to the results obtained when siRNA was applied before infection. These results indicate that it is feasible, at least in the paradigm tested herein, to inhibit replication of TBEV *in vitro* once the infection has been initiated.

For further *in vitro* analysis we developed an encephalitis model based on infection of rat organotypic hippocampal brain cultures with Langat virus. The use of an attenuated virus strain has several advantages compared to the work with human pathogenic TBE strains. Despite its relative avirulence for the human host, LGTV shares a high nucleotide homology with pathogenic TBEV. Nevertheless, LGTV can be handled under BSL-2 conditions. In rodents Langat virus is able to cause viremia, infect the central nervous system and thereby causing encephalitis and neuronal lesions replicating clinical and histopathological features observed in human cases of TBE [36], [43]. Our immunohistochemical results about infection of neuronal cells by LGTV is in agreement with previous findings of fatal TBE human cases where neurons represent the main target for TBEV infection [44]. By measuring virus replication in organotypic hippocampal cultures and staining viral proteins within the neuronal cells we could demonstrate that Langat virus productively infects neurons of rat brain slices (Figure 4C, D, E, F, G, H). In many aspects OHCs fill the gap between dissociated cell cultures and *in vivo* animal experiments. OHCs are ex vivo brain slices and constitute an intact neuronal network with a well-preserved representation of the most important brain cells, including neurons, astrocytes and microglia [45], [46]. This *in vitro* model of neuronal network allows the investigation of the mechanisms underlying neuropathogenicity of TBE and, as we have shown in this study, the efficiency of new antiviral drugs under conditions resembling those encountered *in vivo* [47], [48]. Our results obtained in OHCs transfected with the siRNA sequence D3 support findings from several *in vivo* studies showing that neuronal cells could be transfected with siRNA and are competent to develop a strong siRNA mediated antiviral activity [14], [30], [49], [50]. Applying siRNA sequence D3 on organotypic cell cultures an inhibition of Langat virus replication by more than 99.6% was achieved, both on the level of viral RNA and on the number of infectious particles. After entering the human body, tick-borne flaviviruses develop a high viremia which is considered to be a prerogative for the subsequent invasion of the CNS [51]. The antiviral potential of our siRNA sequences may help to attenuate the viremia and thus prevent the development of encephalitis or, if encephalitis is already established, to limit the number of infected neurons to a minimum thereby minimizing the damage in the brain caused by either the virus itself or the immune response to the infection.

The siRNA sequence D3 targets a well conserved stretch of the 5′UTR of Langat virus sharing 100% homology with all 3 subtypes of TBEV, the European, the Siberian and the Far Eastern subtype as well as with the Omsk hemorrhagic fever virus and 95% (18 nucleotides out of 19) with Louping ill virus (see Table S1). We therefore consider D3 a promising candidate for future *in vivo* studies on TBEV infections. Other siRNA sequences such as D5, D8, D12, D4, Q5 and Q6 were also highly effective in our *in vitro* analysis and may be used in the combination with D3. As it has been suggested in other studies the combination of 2 or more specific siRNAs simultaneously targeting multiple viral genome regions may enhance the inhibitory effect and reduce an eventual viral escape [27], [52].

An unspecific stimulation of the interferon response by the siRNA sequence D3 is unlikely since no upregulation of the IFN-β gene was detected. Cationic lipid reagents, such as Lipofectamine RNAiMAX and Dharmafect 2 used in this study, are promising carriers for the siRNA delivery *in vivo* [53], [54]. However they are known to cause side effects such as cytotoxicity and non-specific activation of intracellular signalling pathways [55], [56]. To determine toxicity of the transfection regimen the LDH release was assessed in several experiments but no increase in LDH level was observed in HeLa cells or in organotypic cell cultures treated with the transfection reagents and siRNA.

Despite these encouraging results, development of reliable transfection methods remains the biggest hurdle in the progress of *in vivo* antiviral RNAi-based drug therapies. In case of encephalitogenic viruses such as TBEV, carriers for therapeutic siRNA molecules must be able to cross the blood-brain barrier [57]. For these reasons the development of new strategies which allow highly efficient transfection of siRNA molecules is a crucial step on the way towards the clinical use of RNAi-based therapies. Organotypic cultures represent a valuable tool to evaluate the efficiency of different transfection strategies with siRNA and to determine their potential to induce the interferon system in a conserved neuronal network.

In summary, our study provides further support for the use of RNAi technology in the development of antiviral drugs against encephalitogenic tick-borne flaviviruses. Organotypic brain cultures were used for the first time as a successful *in vitro* approach in an RNAi-based antiviral therapy.

## Materials and Methods

### Cell Culture and Virus Amplification

Vero and HeLa cells were cultured in Eagle’s minimum essential medium (MEM) supplemented with 10% foetal bovine serum, 1.25% L-Glutamin, 1% Non-essential amino acids, 1% Penicillin-Streptomycin and 0.5% Neomycin-Bacitracin (Biochrom AG, Berlin, Germany). Langat virus strain TP21 was kindly provided by Daniel Růžek (University of South Bohemia, České Budějovice, Czech Republic). Virus was cultured on 80% confluent Vero cells in 75-cm^2^ culture flasks (Sigma-Aldrich, Buchs, Switzerland) and grown for 7 days at 37°C in cell culture medium containing 2% FBS. Virus containing cell culture supernatant was titrated and used for infection and transfection assays.

### Immunoperoxidase Focus Assay (IPFA)

Virus quantification was performed by IPFA. Serial dilutions of virus cultures (100 µl/well) were inoculated on 24 well plates with 80% confluent Vero cells. Viral adsorption was allowed for 1 h at 37°C on a rocking platform and wells overlaid with 200 ul of pre-warmed cell culture medium (2% FBS) and 500 ul MEM containing 1% methylcellulose (Sigma-Aldrich), 5% FBS, 1.25% L-Glutamin, 1% Non-essential amino acids, 1% Penicillin-Streptomycin and 0.5% Neomycin-Bacitracin. Plates were incubated for 6 days at 37°C. Cells were washed with PBS pH 7.4, fixed with 4% formaldehyde in PBS for 1 h and cell membrane permeabilized by adding 1% Triton in PBS for 5 min. Following two washing steps with PBST (PBS +0.05% Tween), 500 µl monoclonal anti-flavivirus antibody (Anti-Flavivirus Group A clone D1-4G2-4-15, Millipore AG, Switzerland) diluted 1∶1000 in blocking buffer (PBST containing 10% FBS and 4% Skimmed Milk powder (Hochdorf Nutritec AG, Sulgen, Switzerland)) was added for 1 h at room temperature (RT). After two washing steps with PBST cells were incubated for 1 h with the secondary goat anti-mouse-HRP antibody (Anti-mouse IgG (H+L); Kirkegaard & Perry Laboratories, Gaithersburg, USA) diluted 1∶1000 in blocking buffer. Viral spots were visualized after thorough washing by adding the HRP substrate AEC (3-amino-9-ethylcarbozole) (Fluka, Switzerland) in N,N-Dimethylformamide (Fluka, Switzerland) diluted in acetic acid 0.05 M (Fluka, Switzerland). Viral titers were determined as focus forming units (FFUs) per millilitre.

### Organotypic Cultures

Organotypic hippocampal cultures were prepared from 5 days-old Wistar rats as previously described by us [33]. Briefly, rat pups were sacrificed by a lethal dose of Pentobarbital i.p (G. Streuli & Cie. SA, Uznach, Switzerland). The brain was removed and submerged in ice-cold dissection medium consisting of Hank’s balanced salt solution (HBSS; Gibco Life Technologies, Basel, Switzerland) with 6 mg/ml glucose and 10 µg/ml Penicillin-Streptomycin. Hippocampus was isolated and cut perpendicular to the axis into 400 µm thick-sections by a McIlwain tissue chopper (Mickle Laboratory, Giuldford, UK). Slices with intact hippocampal morphology were selected and individually transferred on a semiporous (0.4 µm) membrane of the Transwell inserts (Corning Inc., Corning, NY). Inserts were placed in a 24-well plate in contact with 200 µl serum-free Neurobasal ™ medium (Gibco) supplemented with B27 Supplement (20 µl/ml, Gibco). Before transfection, slices were incubated at 37°C with 5% CO_2_ for 4 days. Medium containing B27 Supplement was changed every day for the first two days.

### siRNAs

Six siRNA sequences (Q1–Q6) were designed and synthesized by Qiagen (Hilden, Germany) using the BioPredsi algorithm [58]. Thirteen sequences (D1–D13) (siSTABLE) were designed using the Dharmacon online tool si*DESIGN* ^©^ Center (http://www.dharmacon.com/designcenter) and synthesized by the same company (Thermo Fisher Scientific, Lafayette, USA). SiRNA sequences were chosen according to the algorithm score.

Comparative analysis of all siRNA sequences with genomes of tick-borne flaviviruses was performed using GeniusPro Version 5.5.7 (Table S1).

### Transfection and Infection Assays

Transfection and infection experiments were performed on HeLa cells and organotypic cell cultures. HeLa cells were seeded to a confluency of 60–70% on 24-well plates the day before transfection. When infection of HeLa cells was performed after transfection, cells were first transfected with 200 nM siRNA for 4. SiRNAs were complexed with the transfection reagent Lipofectamine RNAiMAX (Invitrogen, Basel, Switzerland) according to the manufacturer’s instructions and lipid-siRNA complexes added to the cells with 1 ml of serum- and antibiotic-free MEM. After 4 h incubation, cells were washed and infected with 200 µl Langat virus in culture medium at MOI of 10. Virus inoculum was removed after 1 h and cells were incubated with 1 ml antibiotic-free MEM containing 2% FBS for 6 days. Virus titer was assessed by determining the number of genome copies in 100 µl cell supernatant by real-time RT-PCR and number of infectious particles assessed by immunoperoxidase focus assay (IPFA).

When infection was performed before transfection, HeLa cells were infected with Langat virus at a MOI of 10, 1, 0.1 or 0.01. One hour after the infection the inoculum was removed and cells were transfected with 200 nM siRNA/Lipofectamine RNAiMAX complexes as described above. The siRNA/Lipofectamine complexes were removed after 4 hours and cells incubated for 6 days at 37°C. Virus titer was assessed by determining the number of genome copies in 100 µl cell supernatant by real-time RT-PCR 6 days after infection.

Organotypic hippocampal cultures were prepared as described above. After slicing OHCs were cultured for 4 days to allow recovery. OHCs were transfected with 800 nM siRNA complexed with the lipid-based transfection reagent Dharmafect 2 (Thermo Fisher Scientific, Lafayette, USA) for 24 h according to the manufacturer’s instruction. Lipid-siRNA complexes were prepared in 200 µl antibiotic-free Neurobasal Medium (NBM) whereof 100 µl were given to the medium underneath the membrane and 100 µl were added drop wise onto the slice. One day after incubation the OHC were infected with 2×10E6 FFU Langat virus in 200 µl NBM. After 1 h inoculum was removed and OHCs transfected for the second time. Viral genome copy number and number of infectious particles were assessed 2 days after the second transfection step.

### LDH Cytotoxicity Test

HeLa cells transfected with 200 nM siRNA (D3 or nonsense) and untransfected cells were infected with Langat virus 4 h after transfection and incubated for 6 days at 37°C. During 6 days samples of 100 µl supernatant were taken daily and LDH activity was quantified according to the manufacturer’s instructions applying the Cytotoxicity Detection Kit^PLUS^ (LDH) (Roche Diagnostics, Rotkreuz, Switzerland) by measuring optical density (OD) at 490 nm.

### Viral RNA Extraction

Nucleic acid extraction from cell supernatant or OHC homogenate was performed using the BioRobot EZ1 Workstation (Qiagen). 100 µl cell culture supernatant were inactivated in 400 µl AVL viral lysis buffer (Qiagen) and viral RNA isolated applying the EZ1 Virus mini kit v2.0 (Qiagen). OHCs were homogenized in 800 µl Qiazol (Qiagen) by the TissueLyser Sytem (Qiagen) for 2 min at 25 Hz. Extraction was performed with the Universal RNA Tissue Kit (Qiagen) following the manufacturer’s instructions. RNA was eluted in a final volume of 50 µl.

### Primers

The specific primers and probes for the detection of Langat virus strain TP21 were designed using Primer Express software v3.0 (Applied Biosystems, Foster City, CA) and purchased from Microsynth (Balgach, Switzerland). LGTV forward primer 5′-TGTGTGGAGCGGCGATT-3′; LGTV reverse primer 5′-TAAGGGCGCGTTCCATCTC-3′; probe 5′-AGCCACGCTTCCAGAGGAGCACC-3′. Detection of interferon β, OAS-2 and STAT-1 genes was performed using the corresponding pre-designed QuantiTect Primer assays (Qiagen).

### One Step Real-time Reverse Transcriptase PCR

Viral RNA was quantified by one-step real-time RT-PCR using the QuantiFast probe RT-PCR kit (Qiagen) according to the manufacturer’s protocol with the following cycling conditions: reverse transcription at 50°C for 10 min, an initial PCR activation step at 95°C for 5 min, 40 cycles of two-step cycling for 10 s at 95°C and 30 s at 60°C.

### Fluorescence Microscopy

Organotypic hippocampal slices were fixed in 4% paraformaldehyde (PFA) in phosphate-buffered saline for 90 min at 4°C and cryopreserved in 18% sucrose solution in PBS for 3–4 days. Slices were cut into 12 µm thick sections with a Jung CM 1800 cryostat (Leica Microsystems, Glattbrug, Switzerland) according to a procedure described elsewhere [33]. The sections were collected in PBS, transferred to a chrome-alum-gelatine-coated glass slide, air dried and kept in PBS. Organotypic sections were incubated with primary antibodies directed against Langat virus (LGT-I-13A10; 1∶1000 Fort Detrick, USAMRIID, USA) and FOX3 (rabbit polyclonal; 1∶1000, Millipore). Primary and secondary antibodies were diluted in TBS (pH 7.6) containing 0.5% bovine serum albumin. Sections were washed three times with PBS and then exposed to the secondary antibody for 45 min at RT in the dark. Secondary antibodies used were: donkey anti-mouse Cy3 (1∶1000; Jackson, West Grove, PA) and anti-rabbit Alexa Fluor 488 (1∶1000; Invitrogen). After washing slides were counterstained with Dapi for 1 min, washed and mounted with Mowiol® (Merck, Darmstadt, Germany) containing 2.5% Dabco® (Sigma-Aldrich). Stained OHCs were photographed with a Zeiss fluorescent microscope (Axiophot, Zeiss, West Germany).

## Supporting Information

Figure S1 **LDH release of HeLa cells as an index of cytotoxicity over time.** Transfected HeLa cells (siRNA D3 or nonsense siRNA) infected with Langat virus compared to untreated cells infected with Langat virus and to untreated and uninfected cells used as negative control. Cytotoxicity results are expressed as % LDH release of total LDH determined from homogenized HeLa cells used as positive control.
(TIF)

Figure S2 **LDH cytotoxicity assay on transfected organotypic hippocampal cultures.** OHCs transfected twice with siRNA (D3 or nonsense), untransfected OHCs infected with Langat virus or untreated OHCs were tested for cytotoxicity by measuring LDH release in 100 µl of the medium surrounding the slices 48 h after the second transfection. No difference in cytotoxicity was measured between transfected and control groups. Cytotoxicity results are expressed as % LDH release of total LDH determined from homogenized OHCs used as positive control.
(TIF)

Figure S3 **Expression level of IFN-β in transfected OHCs.** Expression levels of IFN-β mRNA was assessed in OHCs treated with siRNA D3 or nonsense siRNA 48 h after the second transfection and compared to IFN-β mRNA levels found in untreated OHCs and in OHCs activated with poly I:C. Data showed that transfection of OHCs did not result in upregulation of IFN-β mRNA expression over the level of untreated slices. The data are presented as the mean ± SD from three independent experiments.
(TIF)

Table S1 **Comparative analysis of siRNA sequences with members of the tick-borne encephalitis virus complex.**
(DOC)
