# Supplementary material for: SiRNA Inhibits Replication of Langat Virus, a Member of the Tick-Borne Encephalitis Virus Complex in Organotypic Rat Brain Slices
Source: PLoS One. 2012 Sep 12;7(9):e44703. doi: 10.1371/journal.pone.0044703 (PMC3440328; doi:10.1371/journal.pone.0044703)
Supplement: Table S1 — Comparative analysis of siRNA sequences with members of the tick-borne encephalitis virus complex. (DOC) [file pone.0044703.s004.doc]

| **Name** | **siRNA sequence** | **Genome region** | **European TBEV subtype**  **(FJ572210.1)** | **Syberian TBEV subtype (AF069066.1)** | **Far Eastern TBEV subtype (JF819648.2)** | **Omsk hemorrhagic fever virus (AY438626.1)** | **Louping ill virus (Y07863.1)** |
| --- | --- | --- | --- | --- | --- | --- | --- |
| Q1 | 5’ – UAUAACGCCCAGUUCGGCCUU – 3’ | 3’UTR | 13/21 | 12/21 | 15/21 | 13/21 | 15/21 |
| Q2 | 5’ – UUGACGGAACGAACCAGGCUG – 3’ | NS3 | 16/21 | 17/21 | 17/21 | 18/21 | 16/21 |
| Q3 | 5’ –UUGAGUUCACUACUCCGGCAU– 3’ | NS1 | 14/21 | 13/21 | 15/21 | 15/21 | 14/21 |
| Q4 | 5’ – UAGUAUGACCAGCCGCCUCUG – 3’ | NS5 | 17/21 | 17/21 | 20/21 | 18/21 | 18/21 |
| Q5 | 5’ – UUAGAUGAUACUUAGUUCCCT – 3’ | Env | 17/21 | 15/21 | 16/21 | 17/21 | 17/21 |
| Q6 | 5’ – UCUGAUGACACUGUGAACGAG – 3’ | Env | 17/21 | 16/21 | 15/21 | 16/21 | 15/21 |
| D1 | 5’ – UUUCUCUCUUCCCUCCUCCUU – 3’ | 3’UTR | 19/19 | 17/19 | 19/19 | 19/19 | 19/19 |
| D2 | 5’ – UCAUCCACAGACUUUGAUCUU – 3’ | NS1 | 18/19 | 16/19 | 15/19 | 16/19 | 17/19 |
| D3 | 5’ – UUUCUCAACACGUUCACGAUU – 3’ | 5’UTR | 19/19 | 19/19 | 19/19 | 19/19 | 18/19 |
| D4 | 5’ – AUGCUCAUGUGUCUUGUCCUU – 3’ | Env | 13/19 | 12/19 | 12/19 | 13/19 | 14/19 |
| D5 | 5’ – UUCUGCAAGGCCUAGUUCCUU – 3’ | Env | 14/19 | 14/19 | 12/19 | 17/19 | 13/19 |
| D6 | 5’ – UUCCACUCCAAUCAUGAACUU – 3’ | NS1 | 13/19 | 12/19 | 12/19 | 10/19 | 11/19 |
| D7 | 5’ – AUAUCCACAAUCACAGUCGUU – 3’ | 5’UTR | 8/19 | 8/19 | 7/19 | 10/19 | 7/19 |
| D8 | 5’ – AUUCCCAGCUCUUGUUCUCUU – 3’ | 5’UTR | 18/19 | 18/19 | 18/19 | 18/19 | 18/19 |
| D9 | 5’ – AAUCCAUCCACCAUCAACCUU – 3’ | pre M | 12/19 | 12/19 | 11/19 | 11/19 | 9/19 |
| D10 | 5’ – UUUCCCGUCACCACUCUCAUU – 3’ | 3’UTR | 17/19 | 18/19 | 18/19 | 17/19 | 16/19 |
| D11 | 5’ – UCUCCCUCGAGCCAUUGGUUU – 3’ | pre M | 16/19 | 18/19 | 18/19 | 17/19 | 16/19 |
| D12 | 5’ – UUUAGAACGGCCUUCCCGGUU – 3’ | Core | 13/19 | 16/19 | 17/19 | 15/19 | 14/19 |
| D13 | 5’ – AAGUCCAUUUGGCAUUUGGUU – 3’ | Core | 18/19 | 18/19 | 18/19 | 18/19 | 18/19 |

**Supporting Table S1: Conservation of siRNA sequences between members of the Tick-borne encephalitis virus complex**
